# Analysis of mRNA and circRNA Expression Profiles of Bovine Monocyte-Derived Macrophages Infected With *Mycobacterium avium* subsp. *paratuberculosis*

**DOI:** 10.3389/fmicb.2021.796922

**Published:** 2022-01-03

**Authors:** Yanhong Bao, Yu Yao, Zi Wang, Shuiyin Wu, Xiuyun Jiang, Hongxia Ma

**Affiliations:** ^1^College of Life Sciences, Jilin Agricultural University, Changchun, China; ^2^College of Animal Science and Technology, Inner Mongolia University for Nationalities, Tongliao, China; ^3^College of Life Sciences, Changchun Sci-Tech University, Changchun, China; ^4^College of Animal Medicine, Jilin Agricultural University, Changchun, China; ^5^The Key Laboratory of New Veterinary Drug Research and Development of Jilin Province, Jilin Agricultural University, Changchun, China; ^6^The Engineering Research Center of Bioreactor and Drug Development, Ministry of Education, Jilin Agricultural University, Changchun, China

**Keywords:** *M*. *avium* subsp. *paratuberculosis*, mRNA, circRNA, high-throughput sequencing, monocyte-macrophage

## Abstract

*Mycobacterium avium* subsp. *paratuberculosis* (MAP) is the pathogen of Johne’s disease (paratuberculosis), which mainly causes chronic infectious granulomatous enteritis in ruminants and has brought huge economic losses to animal husbandry. As a specific intracellular pathogen, when MAP invades the body, it is internalized by macrophages where it is able to replicate by inhibition of the phagosome maturation, escaping the host immune system and surviving, which leads to the spread of the disease. More recent studies have shown that circRNA is involved in many pathological and physiological processes of the body as the molecular sponge of miRNA, the scaffold of RNA binding protein and having the characteristic of being able to translate into protein. In this study, the mRNA and circRNA expression profiles of MAP-infected bovine monocyte-macrophages and uninfected bovine cells were analyzed by high-throughput sequencing. A total of 618 differentially expressed mRNA were screened out, including 322 upregulated mRNA and 296 downregulated mRNA. In addition, the analysis of circRNA differential expression profile showed 39 differentially expressed genes including 12 upregulated and 27 downregulated genes. Moreover, differential genes belonging to cytokine activity, chemokine activity, inflammatory reaction, apoptosis, and other functional groups related to macrophage immune response were significantly enriched in Gene Ontology (GO). Multiple signal pathways including NF-κB, MAPK, Toll-like receptor, IL-17, JAK-STAT, and other signaling pathways related to activating macrophage immune response were significantly enriched in Kyoto Encyclopedia of Genes and Genomes (KEGG). In addition, RT-qPCR technology verified the accuracy of the mRNA sequencing results. In this study, we have obtained the transcriptome information of mRNA and circRNA of bovine monocyte-macrophage infected with MAP. These results will provide data support for the further study of mRNA–miRNA–circRNA network and immune escape mechanism of MAP and will enrich the knowledge of the molecular immune mechanisms of Johne’s disease as well.

## Introduction

*Mycobacterium avium* subsp. *paratuberculosis* (MAP) is the causal agent of Johne’s disease (paratuberculosis). MAP infection can cause chronic granulomatous enteritis, persistent diarrhea, and eventually lead to host death (Wang et al., 2007). The disease is distributed all over the world and mainly infects many ruminants like cattle, sheep, goats, and deer as well as some wild animals (Li et al., 2016). The annual loss of paratuberculosis to American cattle industry is as high as 200 million to 1.5 billion US dollars (Cho et al., 2012). In addition, MAP is also thought to be related to the increase incidence and spread of Crohn’s disease, type I diabetes, sarcoidosis, and other autoimmune diseases in humans (Sechi and Dow, 2015; Slavin et al., 2018).

MAP is transmitted mainly through the fecal–oral route. Calves are infected through contact with contaminated dairy products, soil, manure, water, etc. (Park et al., 2017; Hassan et al., 2020). MAP can also be transmitted through mammary secretions, *in utero*, and via semen (Abbas et al., 2011; Sorge et al., 2013). Vertical transmission of MAP in herds is common, but horizontal transmission has also been observed among domestic animals and between domestic animals and wild animals. In addition, MAP strains can be detected in dust in the living environment of MAP-infected cattle, which further indicates that aerosol is an effective way for its transmission (Eisenberg et al., 2010). During the establishment of persistent infection in macrophages, MAP invade the intestinal tract mainly through M cells in Peyer’s spots and differentiate epithelial cells (Bannantine and Bermudez, 2013). First, MAP is absorbed by M cells, then released in intestinal submucosa and cleared by macrophages (Arsenault et al., 2014; Gupta et al., 2019). Whether the engulfed MAP can be successfully eliminated by macrophages represents a battle between host defense and pathogen proliferation. MAP may play different roles in promoting or inhibiting apoptosis at different time nodes of infecting macrophages (Thirunavukkarasu et al., 2016). For instance, *Mycobacterium tuberculosis* delays apoptosis in the initial stage of infecting macrophages to allow them to replicate in cells, and induces apoptosis in search of new host cells when intracellular conditions are unfavorable for growth (Behar et al., 2010). Detection of mRNA and circRNA expression profiles of host macrophages during MAP infection is helpful to elucidate the pathogenesis of paratuberculosis and the conditional mode of host–pathogen interaction.

At present, circRNA represents a biomarker or regulator of a variety of diseases (Zhang et al., 2018). It is an endogenous, covalently closed circular non-coding RNA. Some circRNA contains miRNA response elements, which can act as a competitive endogenous RNA to bind to miRNA, thereby releasing the inhibitory effect of miRNA on its target genes and upregulating the expression level of target genes. Many studies have shown that circRNA has a certain regulatory position in the diseases of immune, urinary, cardiovascular, and digestive systems (Wang et al., 2017, 2020; Yang et al., 2018; Kolling et al., 2019). Increasing evidence reveals that circRNAs have important roles in the regulation of gene expression at the post-transcriptional level. CircRNAs have been proved to be a key regulatory mechanism of physiological processes such as autophagy and apoptosis (Shi et al., 2020). In addition, Zhang et al. (2017) showed that circRNA plays an important role in the differentiation and polarization of macrophages. Up to now, MAP has not been reported on circRNA expression profile of bovine peripheral blood mononuclear macrophages infected. Therefore, compared with mice and humans, there are still many unsolved mysteries about the role of circRNA in immune regulation and response to external infections such as bacteria in cattle.

Transcriptome is necessary for interpreting genome functional elements and revealing molecular composition in cells and tissues, and plays an important role in understanding organism development and diseases. Macrophages, as the main immune and host cells in the pathogenesis of MAP are vital for control and development of MAP disease. To understand the regulation mechanism of non-coding RNA in MAP-infected bovine mononuclear macrophages, we used high-throughput sequencing technology to study and analyze the mRNA and circRNA expression profiles of MAP-infected bovine peripheral blood mononuclear macrophages.

## Materials and Methods

### Ethics Statement

This study was approved by the Animal Care and Use Committee of Jilin Agricultural University (GB/T 35892-2018, approval on February 6, 2018) and the cattle owner’s consent.

### Monocytes-Derived Macrophages Preparation and Monocytes-Derived Macrophages Infection

The methods for isolation of bovine peripheral blood mononuclear macrophages, obtaining high purity mononuclear cells and infection with bacteria have been described in previous studies (Machugh et al., 2012). Briefly, six healthy Holstein cows (3–6 years old) without Johne’s disease history were selected for this study. Paratuberculosis was proved negative by fecal culture and *IS900* component test. First, the peripheral blood collected from bovine jugular vein was packed into the collection vessel containing anticoagulant sodium citrate. The blood was then slowly added to a sterile centrifuge tube containing Histopaque 1,077 Lymphocyte Separation Solution. After density gradient centrifugation using a horizontal rotor, the brown-yellow layer of peripheral blood mononuclear cells (PBMCs) was collected in a new sterile centrifuge tube. After re-suspending and washing PBMC with phosphate-buffered saline (PBS), red blood cell lysate was used to incubate the cells at room temperature for 5 min to remove contaminated red blood cells. After incubation, the PBMC was washed twice with PBS, the cells were re-suspended with PBS containing 0.5% BSA and 2 mM EDTA, and then monocytes were sorted with MACS magnetic beads. The magnetic beads were selected as anti-human CD14 binding magnetic beads from Meteni Mouse of Germany, and it had been proved that they can cross-react with bovine monocytes (Jacobsen et al., 1993). The CD14-labeled cells remained in the sorting column, and then the CD14-carrying monocytes were eluted from the column for monocytes-derived macrophage (MDM) purification and culture (Supplementary Figure 1A). It was confirmed that the purity of the monocytes finally obtained was ≥ 95% through antibody testing (Supplementary Figure 1B). After differentiation, morphology of monocytes was irregular, and some cells produced antennae (Supplementary Figure 1C).

The cells were prepared in advance in 24-well plates with a cell density of 1 × 10^6^ per well. Then three groups were infected with MAP, the infection multiple was 5:1 while the other three were blank groups. After 6 h of culture, cells were collected, and MDMs were lysed and stored at −80°C for later use until RNA was extracted.

### Library Preparation, Sequencing, and Data Analysis

After taking out the cells that were washed with PBS, the cells were blown by adding lysate until they were fully lysed, and the total RNA of the cells was extracted. The total RNA quantity and purity were analyzed by Bioanalyzer 2,100 and RNA 6,000 Nano LabChip Kit (Agilent, CA, United States) with RIN number ≥ 9.2. Then the cDNA library was constructed and sequenced at both ends.

Using the Illumina paired-end RNA-seq approach, we sequenced the transcriptome, generating a total of million 2 × 150 bp paired-end reads. Reads obtained from sequencing machines include raw reads containing adapters or low quality bases, which affected the following assembly and analysis. Thus, to get high-quality clean reads, reads were further filtered by Cutadapt (version: cutadapt-1.9).^1^ The parameters were as follows: (1) removing reads containing adapters; (2) removing reads containing poly A and poly G; (3) removing reads containing more than 5% of unknown nucleotides (N); (4) removing low-quality reads containing more than 20% of low-quality (*Q*-value ≤ 20) bases. Then sequence quality was verified using FastQC (0.11.9)^2^ including the Q20, Q30, and GC content of the clean data.

The raw data from the mRNA and circRNA sequencing were submitted to the GEO of NCBI, with accession number GSE185609, for the mRNA and circRNA data of the MAP-infected and control groups, respectively.

### Differential mRNA Expression Analysis

Genes with significant differences and stable high expression are likely to participate in important metabolic pathways, perform extremely important biological functions, or work as biomarkers and prognostic indicators for some major diseases.

Two databases, the GO analysis and KEGG pathway, were used to analyze the differentially expressed genes, and Fisher’s test was used to test the significance level of the analyzed results; significance was considered when the *p*-value ≤ 0.05.

### Verification of Differentially Expressed mRNA Real-Time Quantitative PCR

A total of 13 differentially expressed genes were randomly selected from the sequencing results and verified by RT-qPCR. See Supplementary Table 1 for primer information. Reverse transcription and qPCR reaction solution were completed with reagents of Nanjing Nuoweizan Biological Co., Ltd. The RT-qPCR procedure was as follows: 95°C for 30 s, 95°C for 10 s, and 60°C for 30 s. The fold-change of the expression of the transcript mRNA was analyzed using the 2^–ΔΔCt^ method.

### Differential Expression Profile Analysis of circRNA

The differential expression analysis of the identified circRNA was performed using the different multiple and *p*-value. Genes with log2 (FoldChange) absolute value ≥ 1 and *p*-value ≤ 0.05 were required. Then, GO and KEGG pathway were used to analyze the enrichment of differentially expressed circRNA-hosting gene.

## Results

### Sequencing Statistics and Quality Control

The MAP-infected bovine monocyte-macrophage and non-infected bovine monocyte-macrophage were constructed and sequenced. The original read data obtained from the experimental group and the control group were 93.6 million and 102 million, respectively. After removing low-quality sequences, adaptor sequences, and sequences containing more than 5% N (N means that base information cannot be determined), 91.7 and 100 million clean reads were obtained, respectively. The original readings, valid readings, percentages of valid readings, Q20, Q30, and GC contents of each sample are given in Supplementary Table 2. Q20 and Q30 were all above 96%, and the GC content was normal. These data can be used for follow-up analysis.

### Reference Genome Alignment

Tophat was used to compare the pre-processed valid data with the reference genome, which was the *Bos taurus* genome. The reads statistics of the comparison between the sequencing data and the reference genome are shown in Supplementary Table 3. Both the mapping rate and unique mapped rate are above 80%.

### Reference Sequence Density Distribution

**FIGURE 1:**
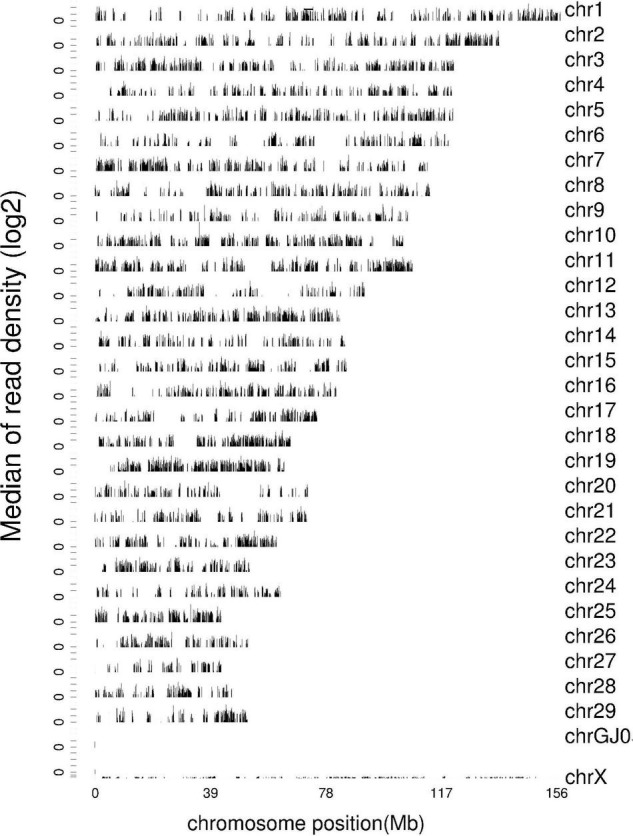
Density distribution map of reference sequence. The abscissa is the position of the chromosome, and the ordinate is the median of read density (log2).

Chromosome density distribution statistics of sequenced sequences located on genome are used to show the distribution of sequenced sequences on chromosomes. The window is 10,000 bp long and maps the coverage depth of each chromosome on the genome; took the median of each base depth in the window range and plotted it with log2 (median) (Figure 1). Under normal circumstances, the longer is the whole chromosome length the more will be the total number of reads located in the chromosome. The relationship between chromosome length and total number of reads can be seen more intuitively from the graph of the relationship between the number of reads located on chromosome along the length.

### Distribution Statistics of Gene Expression Value in mRNA Expression Profile

**FIGURE 2:**
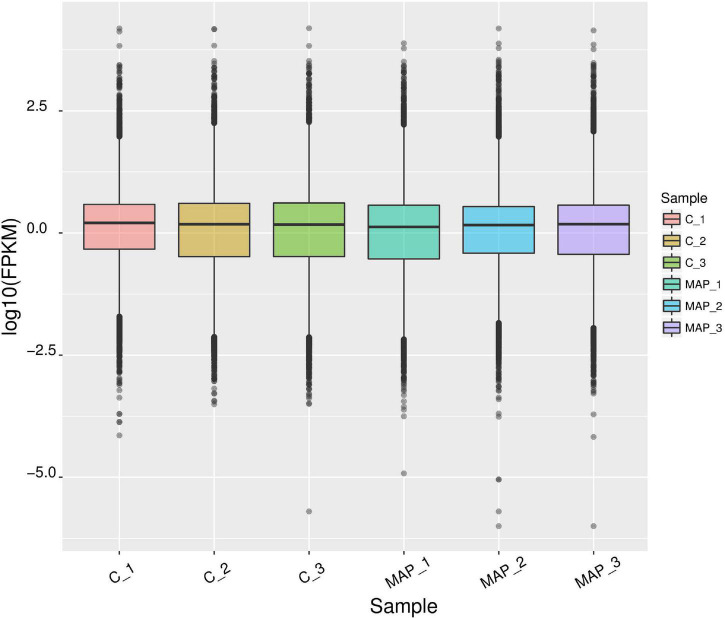
Statistical map of gene expression value distribution of each sample. The abscissa is the sample name, the ordinate is log10 (FPKM), and the box chart of each region corresponds to five statistics (maximum, upper quartile, median, lower quartile, and minimum from top to bottom).

The expression level of genes was measured by FPKM (Fragments Per Kilobase of exon model per Million mapped reads), which can be equivalent to the expression level of genes in different samples. Since the sequencing depth of each sample is different, the absolute gene expression level becomes more after normalization of the FPKM value. The FPKM box diagram of samples can express the gene expression level from the overall level (Figure 2).

### Density of Gene Expression Value in mRNA Expression Profile

**FIGURE 3:**
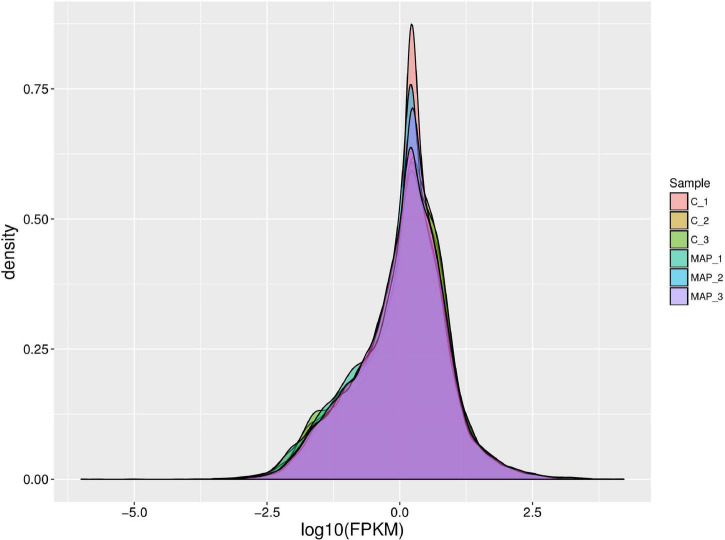
Density map of gene expression values of different samples. The abscissa is log10 (FPKM), and the ordinate is the density of genes.

The expression density map was made by log10 (FPKM) of different samples (Figure 3), and the change of expression trend among different samples could be compared. In an ideal state, the expression density map of each sample should conform to normal distribution, and the expression trend of biological repeated samples should tend to be consistent.

### Analysis of Differential Gene Expression in mRNA Expression Profile

**FIGURE 4:**
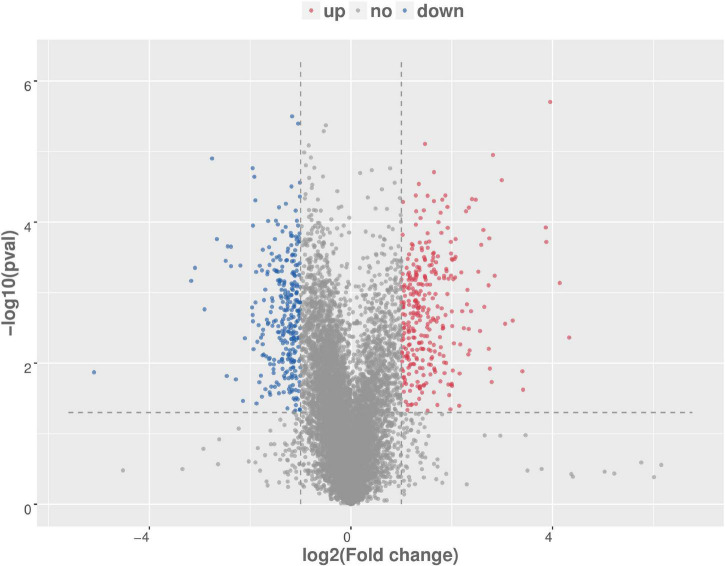
Volcanic map analysis of differential gene expression level. A scatter plot shows the correlation of gene abundance. The red dot, blue dot, and gray dot signify upregulation, downregulation, and not different, respectively.

**FIGURE 5:**
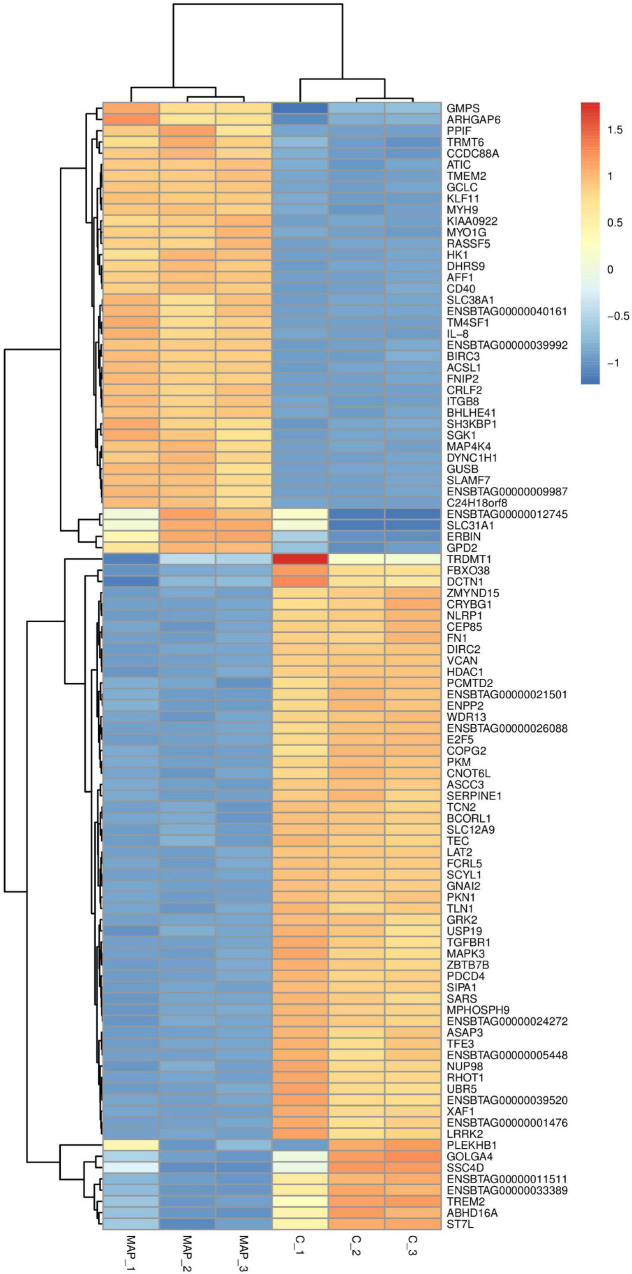
Cluster analysis of differential gene expression level. The abscissa is the sample and the ordinate is the gene. Red indicates high expression gene, and dark blue indicates low expression gene.

There were total of 618 differentially expressed genes (Supplementary Table 4), including 322 upregulated genes and 296 downregulated genes in MAP infected and control MDM cells. Among the upregulated genes, CCL4, CRLF2, NOS2, PPIF, PTGS2, TNFSF9, ACSL1, SPP1, IL10, and TNFRSF18 showed the most significant differential expression. Among the downregulated genes, the ten most significantly expressed genes were HISTIH2BF, CD163, VSIR, CLECI12A, SSH2, FGL2, TNFRSF8, GRK2, EREG, and TGFBR1 (Table 1). To show the overall distribution of differentially expressed genes more intuitively, volcanic maps (Figure 4) and heat maps (Figure 5) were drawn for all genes in differential expression analysis.

**TABLE 1:** The top 20 upregulated and downregulated DE genes (*p*-value ≤ 0.05) for MAP-infected vs. control monocytes-derived macrophage (MDM) samples at 6 hpi as ranked by fold-change.

Gene name	Description	Log2 (fold-change)	*P*-value
CCL4	C-C motif chemokine 4	4.33	0.004335
CRLF2	Cytokine receptor like factor 2	3.95	2E-06
NOS2	Nitric oxide synthase, inducible	3.88	0.000191
PPIF	Peptidylprolyl isomerase F	3.87	0.000119
PTGS2	Prostaglandin-endoperoxide synthase 2	3.21	0.002496
TNFSF9	TNF superfamily member 9	2.85	0.000578
ACSL1	Acyl-CoA synthetase long chain family member 1	2.82	1E-05
PTX3	Pentraxin 3	2.75	0.011946
SPP1	Osteopontin precursor	2.75	0.000170
IL10	Interleukin-10 precursor	2.73	0.006246
HIST1H2BF	Histone H2B type 1	–5.10	0.013466
SAMHD1	SAM and HD domain containing deoxynucleoside triphosphate triphosphohydrolase 1	–3.17	0.000681
CD163	CD163 molecule	–2.91	0.001726
VSIR	V-set immunoregulatory receptor	–2.66	0.000174
CLEC12A	C-type lectin domain family 12 member A	–2.46	0.015198
SSH2	Slingshot protein phosphatase 2	–2.38	0.000223
FGL2	Fibrinogen like 2	–2.28	0.016980
TNFRSF8	TNF receptor superfamily member 8	–2.14	0.034363
GRK2	Beta-adrenergic receptor kinase 1	–1.95	1.72485E-05
EREG	Epiregulin	–1.95	0.002077

### Enrichment and Analysis of Differentially Expressed Genes of Gene Ontology and Kyoto Encyclopedia of Genes and Genomes in mRNA Expression Profile

**FIGURE 6:**
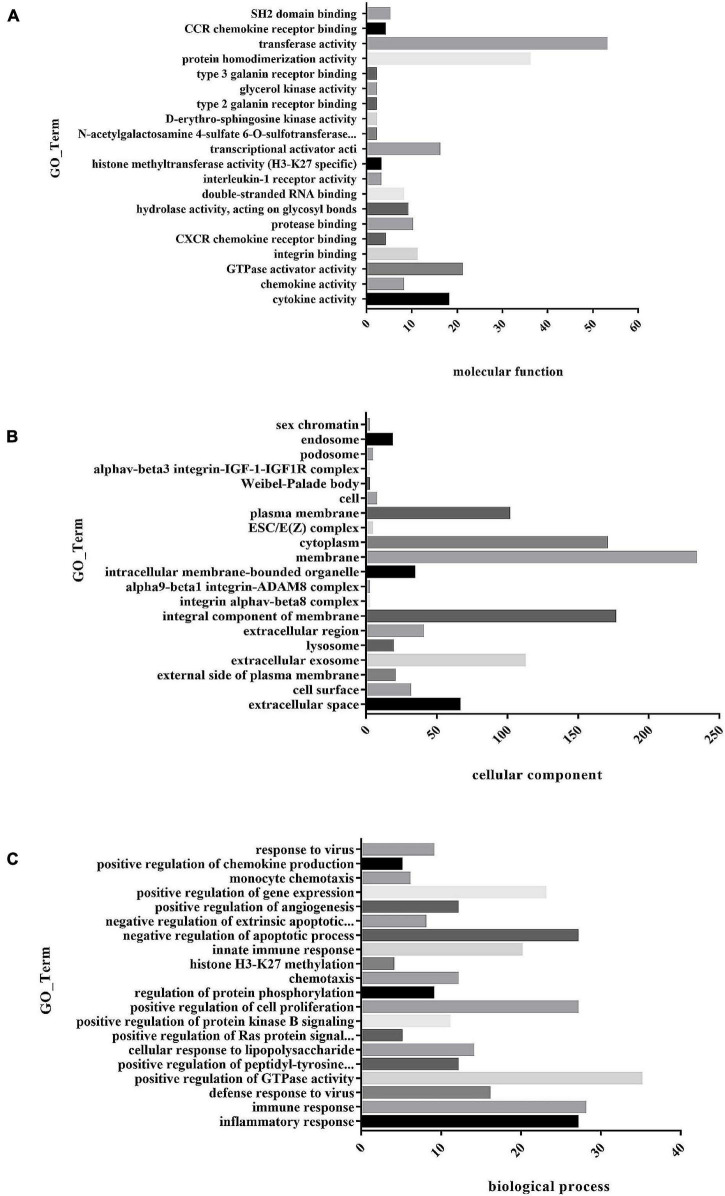
**(A)** Histogram of GO enrichment of differential genes. The first 20 GO enrichment maps of molecular function. **(B)** Histogram of GO enrichment of differential genes. The first 20 GO enrichment maps of cell composition. **(C)** Histogram of GO enrichment of differential genes. The first 20 GO enrichment maps of biological process.

To understand the function of differentially expressed genes in MAP infected bovine monocyte-macrophages, GO and KEGG enrichment analyses were performed on differentially expressed genes. GO analysis of differential genes shows that they were involved in biological processes, cell composition, and molecular function (Supplementary Table 5). In molecular functional classification, the most significant group was cytokine activity (GO: 0005125) with 18 annotated genes, followed by chemokine activity (GO: 0008009) with 8 annotated genes. In the classification of cell components, the most significant functional group was the extracellular space (GO: 0005615) group with 66 annotation genes, followed by the cell surface (GO: 0009986) functional group with 31 annotation genes. In the classification of biological processes, the most significant group was inflammatory response (GO: 0006954) with 27 annotated genes, followed by immune response (GO: 0006955) with 28 annotated genes. In the classification of biological processes, we got a large number of functional groups related to macrophage immune response, such as innate immune response (GO: 0045087) with 20 annotated genes and apoptosis process (GO: 0006915) with 21 annotated genes. Top20 GO Term was selected for the mapping display of molecular functions, cell components, and biological processes (Figures 6A–C).

**FIGURE 7:**
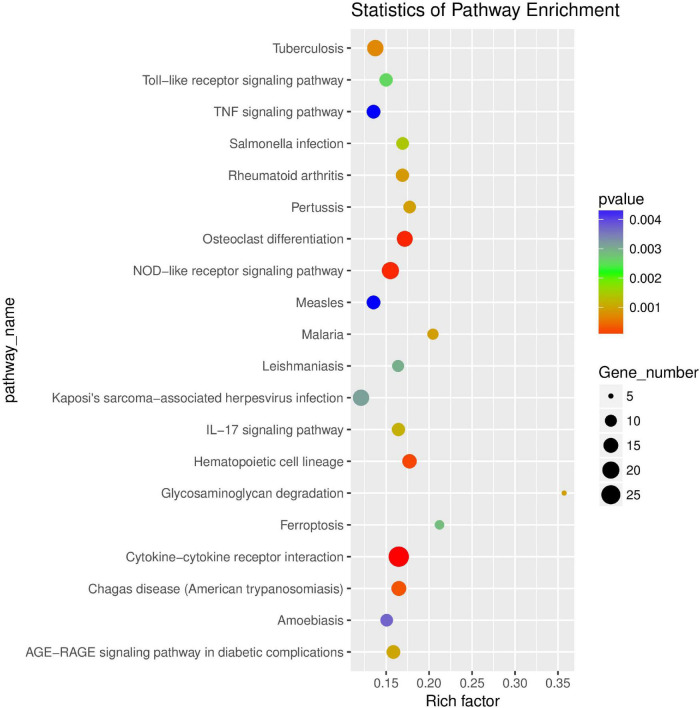
Scatter plot of KEGG enrichment of differential genes. The abscissa Rich factor indicates the number of differential genes located in the KEGG/the total number of genes located in the KEGG. The ordinate is Pathway term, that is, KEGG metabolic pathway.

KEGG enrichment analysis showed that differentially expressed genes were significantly enriched in 53 signaling pathways, including TNF signaling pathway (ko04668), IL-17 signaling pathway (ko04657), and NOD-like receptor signaling pathway (ko04062) (Supplementary Table 6). KEGG enrichment analysis scatter plot (Figure 7) is based on the significance of enrichment by taking the top20 path term to draw.

### Verification of Differentially Expressed Genes in mRNA Expression Profile by RT-qPCR

**FIGURE 8:**
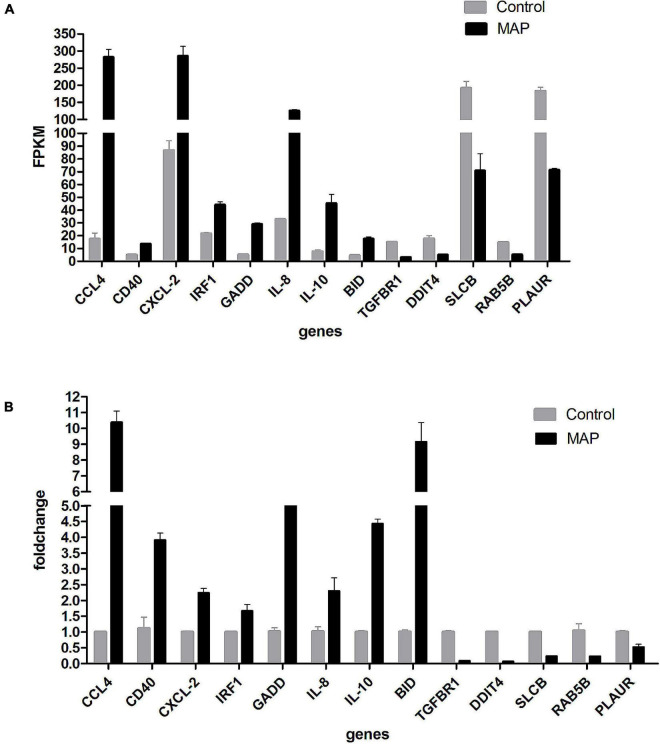
**(A)** RT-qPCR validation. Expression of selected mRNAs validated with RT-qPCR. Drawing with genes as abscissa and FPKM as ordinates. **(B)** RT-qPCR validation. Expression of selected mRNAs validated with RT-qPCR. Drawing with genes as abscissa and fold-change as ordinates.

To verify the accuracy of sequencing results, a total of 13 genes (CCL4, CD40, CXCL-2, IRF1, GADD45B, IL-8, IL-10, BID, TGFBR1, DDIT4, SLCBA5, RAB5B, and PLAUR) were randomly selected for RT-qPCR verification. The results of RT-qPCR were in accordance with the sequencing data, which indicated that our results were reliable (Figures 8A,B).

### Differential Expression Analysis of circRNA

**FIGURE 9:**
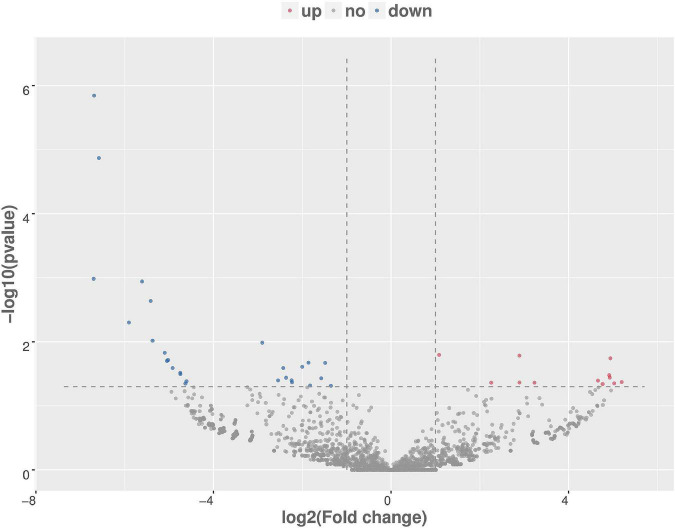
Differentially expressed circRNA volcanic map, with log2 (fold-change) as the abscissa, –log10 (*p*-value) as the ordinate. Red, blue, and gray are representative the upregulated, downregulated, and unchanged circRNAs, respectively.

**FIGURE 10:**
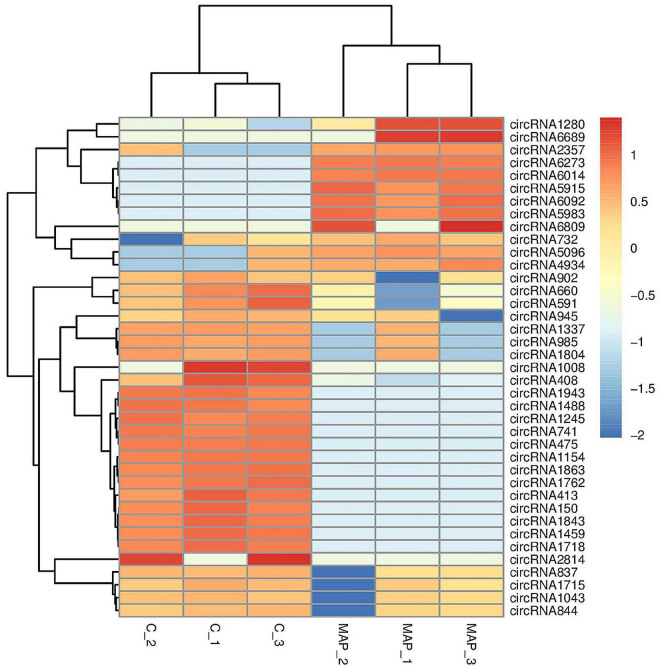
Cluster analysis of differentially expressed circRNA. The abscissa is the sample, and the ordinate is the differentially expressed genes screened out. Red indicates highly expressed genes, and dark blue indicates low expressed genes.

The number of upregulated and downregulated significantly differentially expressed circRNAs was counted in the experimental group and the control group, and there were 39 differentially expressed circRNAs (see Supplementary Table 7 for details). Among them, the frequency of upregulated genes was 12, and the frequency of downregulated genes was 27 (Figure 9). Among them, 20 upregulated genes and downregulated genes with the most significant differences corresponding to circRNA are shown in Table 2. In addition, we performed a cluster analysis of the differentially expressed circRNA genes to more intuitively display the differential expression of genes between the MAP infection group and the control group, as shown in Figure 10.

**TABLE 2:** The top 20 upregulated and downregulated DE genes (*p*-value ≤ 0.05) for MAP-infected vs. control MDM samples at 6 hpi as ranked by fold-change.

circRNA ID	Log2foldchange	*p*-value	chr	Gene name
circRNA6809	5.192305115	0.042347	chr13	PLXDC2
circRNA6689	5.027111833	0.044368	chr9	CRYBG1
circRNA6273	4.940363769	0.018076	chr29	CRLF2
circRNA5915	4.925653435	0.036017	chr15	CD44
circRNA6092	4.913447368	0.033156	chr24	WDR7
circRNA5983	4.765632834	0.045534	chr19	TEX2
circRNA6014	4.66131962	0.040178	chr18	FTO
circRNA2357	3.232074607	0.0432925	chr10	LVRN
circRNA4934	2.893491156	0.043064	chr28	GPR137B
circRNA5096	2.892591233	0.016405	chr5	PLXNC1
circRNA1008	–6.70168	0.001035	chr23	PRIM2
circRNA1154	–6.69119	1.43E-06	chr28	TT+Z2:Z40C13
circRNA1863	–6.58103	1.35E-05	chr1	BRWD1
circRNA2814	–5.90587	0.004991	chr27	PLEKHA2
circRNA1459	–5.6113	0.00114	chr5	FGD4
circRNA1943	–5.41418	0.002293	chr9	ERMARD
circRNA413	–5.37358	0.00959	chr10	ATP8B4
circRNA1245	–5.09834	0.014819	chr7	MYO1F
circRNA150	–5.04872	0.019825	chr12	ZC3H13
circRNA1762	–5.02587	0.019243	chr2	UBR4

### Gene Ontology and Kyoto Encyclopedia of Genes and Genomes Enrichment Analysis of Differentially Expressed circRNA-Hosting Gene

**FIGURE 11:**
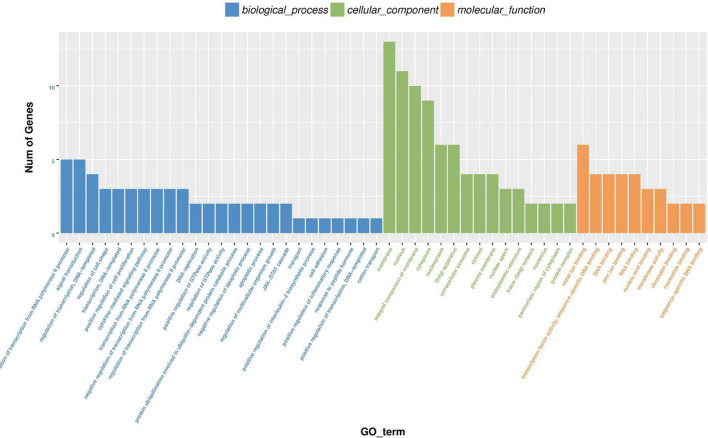
Histogram of differential expression circRNA-hosting gene GO enrichment analysis. Take GO term as abscissa and Num of genes as ordinate.

For the function of circRNA in bovine mature body, we carried out enrichment analysis of GO and KEGG, respectively. The results of GO enrichment analysis are shown in Supplementary Table 8. According to the descending order of S gene number (the number of genes with significant differences annotated as a specific GO) from large to small, GO Term of Top25, Top15, and Top10 were selected for mapping display for biologic process, cellular component, and molecular function, respectively (Figure 11).

**FIGURE 12:**
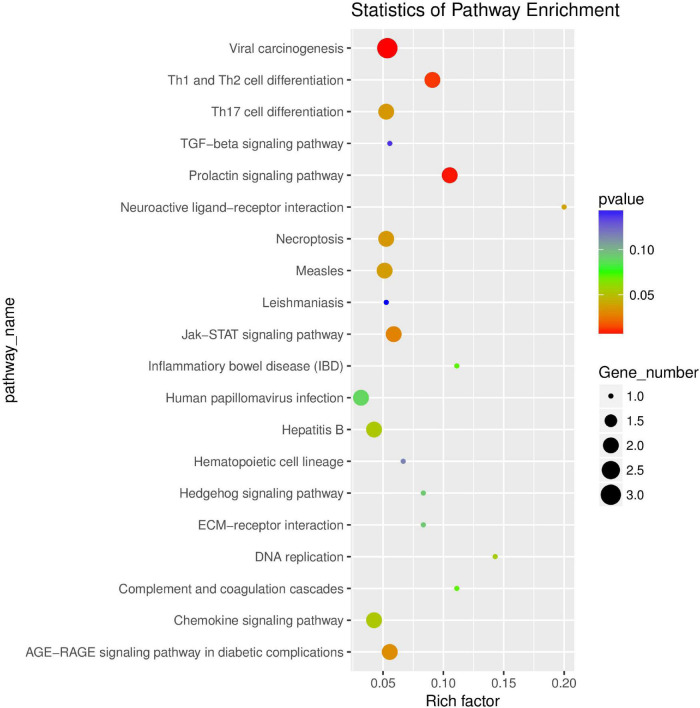
Scatter plot of differential expression circRNA-hosting gene KEGG enrichment analysis. The abscissa is Rich factor (Rich factor = S gene number/B gene number). The ordinate is Pathway term, that is, KEGG metabolic pathway.

The results of KEGG enrichment analysis are shown in Supplementary Table 9. Pathway term of Top20 was selected for mapping according to *p*-value of enrichment (Figure 12). KEGG enrichment analysis showed that it was significantly enriched in JAK-STAT signaling pathway, Th17 cell differentiation, Necroptosis, Th1 and Th2 cell differentiation, and chemokine signaling pathway.

## Discussion

Among the four subspecies of *M. avium*, MAP is the most threatening to animal husbandry, and the chronic wasting enteritis caused by MAP can cause intractable diarrhea and progressive emaciation in ruminants (mainly cattle). Although it has been reported that *M. avium* subsp. *avium* and *M. avium* subsp. s*ilvaticum* can be isolated from cattle (Matthews and McDiarmid, 1979; Turenne et al., 2006); they normally do not cause a large-scale outbreak, which is why MAP was selected for cell infection test in this part of the study.

At present, the continuous improvement of high-throughput transcriptome sequencing technology and the increasingly complete gene bank data provide powerful basis for the understanding of the gene expression of macrophages in response to bacterial infections. More specifically, we can deeply understand the complex interaction process between host animal macrophages and mycobacterium pathogens, thus providing a basis for understanding the molecular mechanism of mycobacterium-induced diseases. However, most of the current studies have focused on the differences in host gene expression between humans and *Mycobacterium bovis* infections, and there is a lack of studies on each subspecies of *M. avium*. Therefore, transcriptome analysis of bovine monocyte-macrophages infected by MAP was performed in our study, which aims to provide a theoretical basis for clarification of immune response mechanism of macrophages in the early stage of *M. avium* infection.

After 6 h of infection, CCL4 gene, an important inflammatory chemokine, increased most obviously in monocyte-macrophage mRNA differential expression. CCL4 binds to its major receptor G protein-coupled receptors CCR5 and CCR8, and can initiate the migration of immune cells, the maturation of dendritic cells, and the activation and differentiation of granulocytes and T cells (Castellino et al., 2006). In addition, the expression of other pro-inflammatory factors or chemokines like CCL2, CCL20, CXCL2, and IL-1β is also significantly upregulated. These gene products play an important immunomodulatory role in the early stages of mycobacterial infection (Bannantine and Bermudez, 2013). Furthermore, we also pay attention to the significant upregulation of CD40 gene encoding TNF receptor super family protein member 5 expressed on the surface of many cells including B cells, macrophages, and dendritic cells. By binding with CD40 ligands on T cells, T cells are activated to activate macrophages, and then induce genes encoding IL12β and NOS (Guida et al., 2011). It has been reported that CD40 knockout mice infected with *M. avium* show effects on IL-12 and IFN-γ, which indicates that CD40 makes an important impact on the development of immune response caused by mycobacterium infection.

It is worth noting that the expression level of IL-10 gene also increased by nearly 7 times. CD4^+^ T cells and monocytes/macrophages are the most important sources of IL-10. IL-10 functions to inhibit the anti-mycobacterial activity of macrophages with possible mechanism of action to limit cytokine-induced tissue damage and inflammatory response during infection by inhibiting the activity of NF-κB signaling pathway in cells (Romano et al., 1996; Haddad et al., 2003). At the same time, it has been reported that phosphorylation of MAPK14 (p38-α) is the main inducement leading to upregulation of IL-10, which leads to subsequent inhibition of host innate immune response and enhancement of *Mycobacterium intracellulare* proliferation (Elass et al., 2008). It is worth noting that Weiss et al. (2008) reported that the expression of IL-10 in human monocytes was inhibited after infection with MAP, which was contrary to the results of this experiment. Simultaneously, the expression of MAPK14 was found significantly downregulated after infection. The effect may be due to the non-detection of post-transcriptional modification of MAPK14 (such as phosphorylation), or IL-10 expression may be induced by another cellular signaling mechanism. The mitogen-activated protein kinase (MAPK) signaling cascade is made up of P38, JNK, and ERK (Reiling et al., 2001). Expression of IL-10 is mediated through activation of MAPKp38, and is independent of MAPK-ERK or MAPK-JNK. Moreover, according to the study of Weiss et al., the phosphorylation of MAPKp38 induced by MAP may be mainly through the interaction with TLR2 cell membrane receptor. In the early stage of MAP infection of bovine mononuclear macrophages, pathogen interacts with the TLR2 cell membrane receptor and causes the phosphorylation of MAPKp38, resulting in the increase of IL-10 in bovine monocyte-macrophages (Souza et al., 2008). This process occurs faster than transcription of MAPK14 gene. Therefore, in the early stage of MAP infection, the increase in IL-10 mRNA expression is not directly related to the decrease in MAPK14 mRNA expression. IL-8, which can enhance the clearance of MAP bacteria by macrophages, is also significantly upregulated. According to Coussens et al. (2004) the expression levels of IL-10 and IL-8 in PBMC, intestinal lesions, and mesenteric lymph nodes of cattle naturally infected with MAP were significantly higher than the control group, which was consistent with the results of this experiment. In addition, MAP can activate NOD-like receptor, and then activate NF-κB and MAPK signaling pathway, thus promoting the expression of IL-8. Therefore, the increase in IL-10 mRNA expression is not directly related to the increase in IL-8 mRNA expression. This mutual neutralization also reflects the characteristics of mycobacterium infecting macrophages.

Previous studies have emphasized the importance of apoptosis in the process of host infection with mycobacteria. Macrophage apoptosis is considered to be a host innate immune mechanism to control mycobacterial infection by limiting growth. At the same time, mycobacteria can delay apoptosis in the early stage of infection of macrophages to allow them to replicate in cells (Behar et al., 2011). By GO enrichment analysis, a total of 21 genes related to apoptosis were found significant differentially expressed, of which 15 genes were upregulated and 6 genes were downregulated. PPIF, the most upregulated gene, has been reported that its coding product cyclophilin-D (CycD) is a key component of the mitochondrial permeability transition pore (mPT) that can regulate cell apoptosis independently of BCL-2 (Speir et al., 2017). Moreover, the expression of TNF, CASP6, BIRC3, and other apoptosis-related genes showed an upregulated trend although there was no significant difference. The specific role of these apoptosis genes in the process of MAP infection needs further study and confirmation.

In the analysis of differentially expressed gene pathways using KEGG database, a number of signal pathways related to activating host immune response to mycobacterium were enriched. Macrophage recognition of MAP is mediated by host pathogen recognition receptors, including Toll-like receptors (TLRs) and NOD-like receptors (NLRs) (Delbridge and O’Riordan, 2007; Killick et al., 2013). Based on our results, there are 12 and 20 significant differentially expressed genes annotated to Toll-like receptor and NOD-like signaling pathway, respectively. Previous studies have reported that TLR2 plays an important role in early recognition of *M. avium* by macrophages, and it has been reported that TLR2 is upregulated after MAP bacteria infect macrophages for 2 h (Machugh et al., 2012); however, no significant difference in TLR2 expression after 6 h of infection was observed in this experiment. MAPK signaling pathway consists of evolutionarily conserved serine/threonine kinase, which involves a variety of cell functions, including cell proliferation, differentiation, and migration. MAPK signaling pathway modulates the expression of inflammatory chemokines and cytokines in innate immune responses such as IL-1, IL-10, IL-12, and TNF-α by transmitting stimuli to transcription factors (Zhang and Dong, 2005). There are multiple genes annotated in MAPK signaling pathway where differentially expressed genes showed some opposite trends. As described earlier, MAP3K14 was downregulated, while MAP4K4 was upregulated, and FOS and SP1 also showed downregulated trends. Besides, many signaling pathways such as NF-κB, IL-17, p53, and cytokine–cytokine receptor interaction were significantly enriched.

The expression profile of circRNA after MAP infects bovine monocyte-macrophages was also analyzed to provide a theoretical basis for the pathogenesis of paratuberculosis and to find potential circRNA biomarkers in paratuberculosis. In this experiment, a total of 39 differentially expressed circRNA were detected, of which 12 were upregulated and 27 were downregulated. The differentially expressed circRNA were all derived from exons. Several differentially expressed circRNAs exist on chromosomes 18 and 19. It is well accepted that most of the genes on chromosomes 18 and 19 are related to immune response signaling pathways, and most are involved in multiple immune pathways including chemokine signaling pathways and NOD-like receptor signaling pathways (Gupta et al., 2019). The scatter plot of differentially expressed circRNA-hosting gene KEGG showed that the differentially expressed genes were significantly enriched in the following pathways: Th1 and Th2 cell differentiation, JAK-STAT signaling pathway, necroptosis, Th17 cell differentiation, and chemokine signaling pathway. Cytokines such as IFN-α, IFN-γ, IL-2, GM-CSF, and TNF-α produced by Th1 cells participate in cell-mediated immunity against intracellular bacterial and viral pathogens; Th2 cytokines such as IL-4, IL-5, IL-6, IL-10, IL-13, and TGF-β produced by Th2 cells play a role in enhancing humoral immunity and resisting extracellular bacteria, parasites, toxins, and allergens. Th17 cells, as a subset of CD4^+^ T cells, are classified as inflammatory Th subgroups, which can cause chronic tissue inflammation and organ failure. It relates to the pathogenesis of most common autoimmune diseases. Besides IL-17, Th17 cells also express a series of inflammatory cytokines, including IL-17F, IL-26, IL-21, IL-22, GM-CSF, and inflammatory chemokine (CC motif) ligand 20 (CCL20). Therefore, the balanced differentiation of Th17 cells is very important for immunity and host protection; JAK-STAT signal mediates almost all immune regulation processes and is a universal and important way of cytokine receptor signal transduction. This pathway is involved in many important biological processes such as cell proliferation, differentiation, apoptosis, and immune regulation. Various studies have shown that the continuous activation of the JAK/STAT signaling pathway is closely related to many immune and inflammatory diseases. Necrosis and apoptosis are two different ways of cell death. Apoptosis is a programmed cell death, while necrosis is mainly a regulatory death method mediated by RIPK1, RIPK3, and MLKL. In short, necrosis has been shown to be involved in the pathogenesis of various diseases. However, under certain circumstances, necrosis may benefit host’s resistance to pathogen infection. CircRNAs may inhibit or promote gene expression. However, their role remains to be verified. The potential role of circRNAs as a biomarker has been proven, and it may also become a promising method for the diagnosis of paratuberculosis. Furthermore, whether circRNA can be used as a potential biomarker to determine MAP-specific infection remains to be discussed.

In conclusion, a large amount of biological information including multiple genes with significant differences and multiple cell signaling pathways responding to infection were obtained by transcriptome sequencing. The aforementioned results contribute to clarify the key cell pathways involved in the early stages of infection. Screening of gene expression profile related to infection can also provide a theoretical basis for diagnosis improvement of MAP. In addition, although some differential genes may not be clearly related to paratuberculous infection, they still provide new insights into pathogenesis of MAP or unknown defense strategies of host cells.

## Data Availability Statement

The data presented in the study are deposited in the NCBI (https://www.ncbi.nlm.nih.gov/geo/query/acc.cgi?acc=GSE185609) repository, accession number GSE185609.

## Author Contributions

YB, XJ, and HM conceived the research. YB wrote the article. YY, ZW, and SW critically reviewed the findings and improved the article. All authors contributed to the article and approved the submitted version.

## Conflict of Interest

The authors declare that the research was conducted in the absence of any commercial or financial relationships that could be construed as a potential conflict of interest.

## Publisher’s Note

All claims expressed in this article are solely those of the authors and do not necessarily represent those of their affiliated organizations, or those of the publisher, the editors and the reviewers. Any product that may be evaluated in this article, or claim that may be made by its manufacturer, is not guaranteed or endorsed by the publisher.

## Footnotes

1
https://cutadapt.readthedocs.io/en/stable/

2
http://www.bioinformatics.babraham.ac.uk/projects/fastqc/

## Supplementary Material

Supplementary Figure 1 **(A)** Bovine peripheral blood mononuclear cells sorted by MACS. **(B)** The result of purity of CD14^+^ cells. **(C)** Monocytes that partially produce antennae.

Supplementary Table 1 RT-qPCR primer sequence list.

Supplementary Table 2 Sequencing sequence statistics and quality control results.

Supplementary Table 3 Reference genome comparison statistics.

Supplementary Table 4 Differential mRNA expression results.

Supplementary Table 5 Enrichment and analysis of differentially expressed genes GO.

Supplementary Table 6 Enrichment analysis of differential gene KEGG.

Supplementary Table 7 circRNA differential expression analysis.

Supplementary Table 8 circRNA GO enrichment analysis results table.

Supplementary Table 9 KEGG enrichment analysis results table.
