# Supplementary figures and images for: Analysis of mRNA and circRNA Expression Profiles of Bovine Monocyte-Derived Macrophages Infected With Mycobacterium avium subsp. paratuberculosis
Source: Front Microbiol. 2022 Jan 3;12:796922. doi: 10.3389/fmicb.2021.796922 (PMC8761944; doi:10.3389/fmicb.2021.796922)

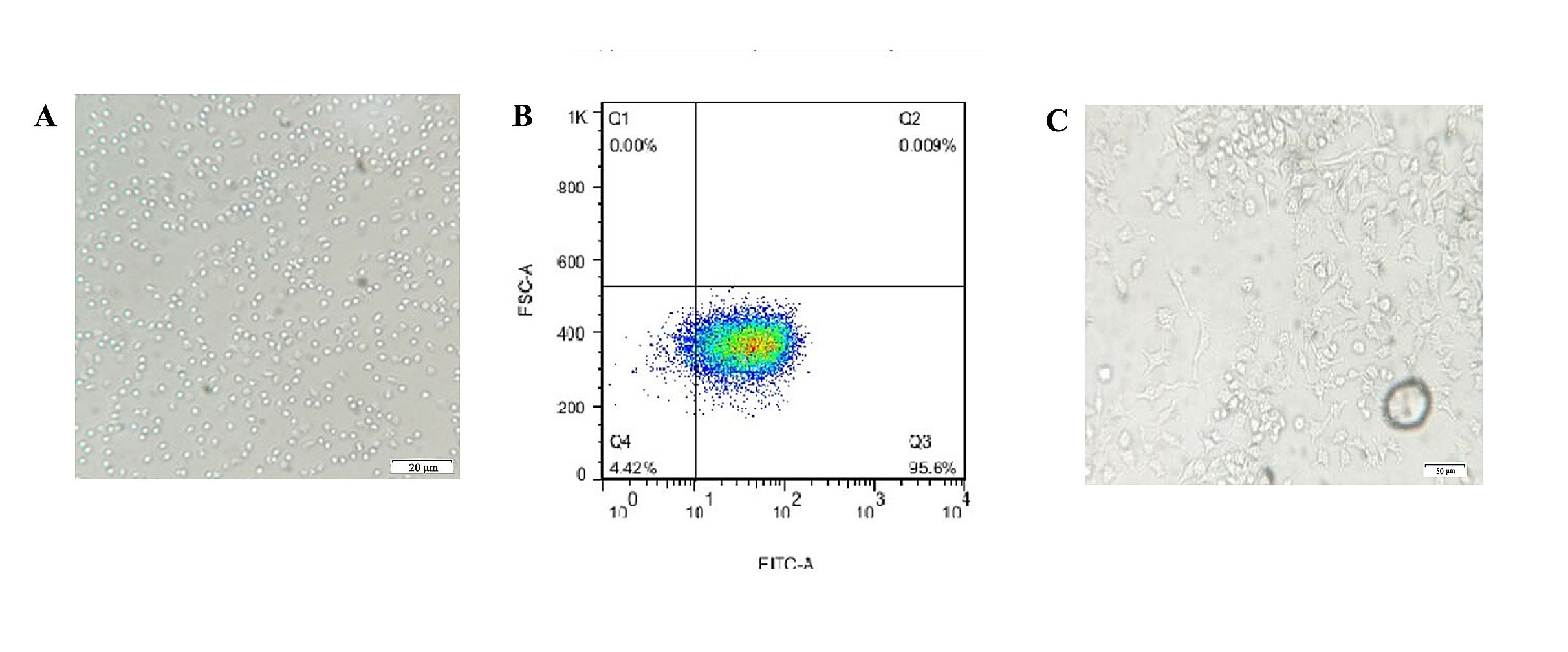

Supplement: Supplementary Figure 1 — (A) Bovine peripheral blood mononuclear cells sorted by MACS. (B) The result of purity of CD14+ cells. (C) Monocytes that partially produce antennae. [file Image_1.JPEG]
